# Pyoderma Gangrenosum Can Now Be Diagnosed Reliably with Validated Scores

**DOI:** 10.1007/s00266-025-05227-7

**Published:** 2025-09-22

**Authors:** Joachim Dissemond

**Affiliations:** Department of Dermatology, Venerology and Allergology, University of Essen, Hufelandstr. 55, 45147 Essen, Germany

*Level of Evidence II* This journal requires that authors assign a level of evidence to each article. For a full description of these Evidence-Based Medicine ratings, please refer to the Table of Contents or the online Instructions to Authors www.springer.com/00266.

Dear editors,

I read the article by Dahy et al., “Outcomes and risk factors of pyoderma gangrenosum after reduction mammoplasty: a systematic review of the literature and case report,” with great interest [1]. Through their systematic review, the authors make a valuable and important contribution to raising awareness of this rare but often severe postoperative complication.

There is, however, one aspect concerning the diagnosis of pyoderma gangrenosum (PG) that merits further emphasis. The authors rightly state that diagnosis is challenging and that misdiagnoses are common. This is absolutely correct [2]. In this context, it is important to highlight that two validated diagnostic scoring systems for PG have now been established. These tools are straightforward to apply and should be familiar, or made familiar, to surgical colleagues.

Among these, the PARACELSUS score currently offers the highest diagnostic accuracy for identifying PG [3, 4]. Therefore, in cases of postsurgical wound healing disorders following reduction mammoplasty that present atypically or are unresponsive to antibiotic therapy, this score should be employed at an early stage to support or confirm the diagnosis of PG. Early recognition using validated diagnostic tools is essential to initiate appropriate therapy promptly and thereby prevent serious complications for affected patients.
